# Cave features, seasonality and subterranean distribution of non-obligate cave dwellers

**DOI:** 10.7717/peerj.3169

**Published:** 2017-05-10

**Authors:** Enrico Lunghi, Raoul Manenti, Gentile Francesco Ficetola

**Affiliations:** 1 Department of Biogeography, Universität Trier Fachbereich VI: Raum-und Umweltwissenschaften, Trier, Germany; 2 Museum of Natural History of Florence—Zoology Section (“La Specola”), University of Florence, Firenze, Italy; 3 Natural Oasis, Prato, Prato, Italy; 4 Departement of Environmental Science and Policy, Università degli Studi di Milano, Milano, Italy; 5 Laboratoire d’Ecologie Alpine (LECA), Université Grenoble-Alpes, Grenoble, France; 6 LECA, Centre National de la Recherche Scientifique (CNRS), Grenoble, France

**Keywords:** Biospeleology, Amphibian, Spider, Cave biology, Invertebrate, Gastropoda, Detection probability, Cricket, Community, Mixed models

## Abstract

Seasonality impacts species distributions through changes of the environmental conditions that affect the presence of individuals at a given place. Although the dynamics of cave microclimates are well known, only a few studies have evaluated the effects of such dynamics on non-strictly cave species. Here we assessed if species exploiting subterranean environments show changes in habitat occupation related to seasonal variation of cave microclimates. We surveyed 16 caves in central Italy every month for one year. Caves were subdivided into longitudinal sectors of three meters. In each sector we measured cave morphology and microclimatic features, assessed the occurrence of eight non-troglobitic taxa (orthopterans, spiders, gastropods and amphibians), and related species distribution to environmental features and sampling periods. The occurrence of most species was related to both cave morphology and microclimatic features. The survey month was the major factor determining the presence of species in cave sectors, indicating that cave-dwelling taxa show strong seasonality in activity and distribution. For multiple species, we detected interactions between sampling period and microclimatic features, suggesting that species may associate with different microhabitats throughout the year. The richest communities were found in sites with specific microclimates (i.e., high humidity, warm temperature and low light) but seasonality for species richness was strong as well, stressing the complexity of interactions between outdoor and subterranean environments.

## Introduction

Seasonality plays a major role in species distributions by altering the presence of individuals in a given place at a stated time (Murray, Webster & Bump, 2013). Seasonality may also impact other biological functions of individuals, such as growth, feeding, and reproduction (Araújo et al., 2010; Hjernquist et al., 2012). Among climatic features, air temperature and water availability have a particularly strong impact on species phenology (Kearney et al., 2013), principally on ectotherms (Amarasekare & Coutinho, 2014; Sheldon & Tewksbury, 2014), and variation in these features often force animals to search for environments with the most suitable conditions (Papaioannou et al., 2015; Seebacher & Alford, 2002).

Subterranean environments, from small crevices to deeper holes and caves, are sometimes used as shelters to avoid unfavorable outdoor conditions (Biswas, 2014; Lunghi, Manenti & Ficetola, 2014; Manenti, 2014), as these environments possess specific microclimatic features which differ from those of surface habitats (Romero, 2009). However, even in such environments the microclimate may not be stable, and fluctuations of primary microclimatic features (temperature, humidity, illuminance) contribute to creating areas characterized by heterogeneous conditions, especially in zones not far from the surface (Campbell Grant, Lowe & Fagan, 2007; Culver & White, 2005; Romero, 2012). Species inhabiting such zones are mainly ascribed to troglophiles and trogloxenes, two categories of non-strictly cave species that are able to leave subterranean environments (Sket, 2008). Nevertheless, such species do not occur by chance in subterranean environments, as they need specific combination of environmental features. Consequently, these species tend to occupy zones in which preferred microclimate conditions are realized (Lunghi, Manenti & Ficetola, 2014). However, only a few studies have evaluated the effect of cave microclimatic dynamics on non-strictly cave species (Camp et al., 2014; Lunghi, Manenti & Ficetola, 2015; Mammola, Piano & Isaia, 2016), leaving incomplete knowledge about species-habitat associations across seasons.

In this study we investigated if a group of non-strictly cave species inhabiting subterranean environments changes its distribution through seasons accordingly to cave microclimatic changes. We focused on non-strictly cave species as such species are often considered to use subterranean environments occasionally, and consequently they are likely linked to the characteristics of both outdoor and subterranean environments. Specifically, we investigated (a) the occurrence of individual species and (b) if the total richness of taxa in a subterranean phase showed stable habitat associations across the year, identifying general patterns and idiosyncrasies of species responses.

## Materials & Methods

### Surveys

We surveyed 16 caves in the Northern Tuscan Apennines (Central Italy, between 43°52′29″N, 11°13′04″E and 43°59′51″N, 10°13′48″E) monthly from January to December of 2013. We completely explored 13 sites, while the remaining three caves were explored until the point at which speleological equipment was indispensable and exploration became too difficult (minimum explored length = 6 m, maximum = 60 m, average ± SE = 23.44 ± 3.94 m). All caves were divided into sectors of 3 m in length, which resulted in 125 cave sectors, considering all the caves (Table S1). We decided to divide the inner environment into three meters cave sectors, as this subdivision allows adequate characterization of microclimatic variation within caves (Lunghi, Manenti & Ficetola, 2015). During each survey, we recorded morphological and environmental features for each sector. On a few occasions, some sectors were not accessible because of temporary inaccessibility (e.g., flooding) and missing data represents 2.7% of the observations. At the end of each sector, we measured maximum height and width using a metric wheel. Furthermore, we estimated average wall heterogeneity (i.e., richness of clefts; Camp & Jensen, 2007) by placing a string of 1 m in the middle of the sector and measuring the distance between the two string extremities using a measuring tape. We performed the measures for both right and left walls and calculated the average (Lunghi, Manenti & Ficetola, 2014). Air temperature and humidity were recorded using a Lafayette TDP92 thermo-hygrometer (accuracy: 0.1 °C and 0.1%) adopting precautions to avoid influence on cave microclimate (Lopes Ferreira et al., 2015), while the average incident light of sectors was estimated using a Velleman DVM1300 light meter (minimum recordable light: 0.1 lux).

### Detection of species

We performed 12 monthly surveys in each cave and the interval between two consecutive surveys in the same cave varied between nine and 45 days, to have surveys for every month of the year. All 3 m-sectors were re-sampled by the same person, who dedicated 7.5 min to each sector to assess the presence of species using visual encounter surveys (Crump & Scott, 1994).

During the surveys, we assessed the presence and distribution of eight trogloxenic and troglophilic taxa within each cave sector: one orthopteran (*Dolichopoda laetitiae*), three spiders (*Meta menardi*, *Metellina merianae*, and *Tegenaria* sp.), two gastropods (*Chilostoma planospira* and *Limax* sp*.*) and two anuran amphibians (*Rana italica* and *Bufo bufo*). The species were selected due to their common presence in caves and their easy identification without invasive methods, giving us the opportunity to collect data limiting disturbance and changes in individual behaviour. Furthermore these species show significant variation in life history and ecological traits. The cave cricket *Dolichopoda laetitiae* is generally abundant in subterranean environments of Central Italy (Allegrucci et al., 2014). These crickets are scavengers of subterranean environments but can also feed outside caves, thus increasing the supply of allochthonous organic matter into the caves (Lavoie, Helf & Poulson, 2007; Weckerly, 2012). The three arachnid species exploit subterranean environments for a significant part of their lives and are among the major cave predators (Manenti, Lunghi & Ficetola, 2015; Novak et al., 2010). We grouped all detected individuals as *Tegenaria* sp*.* because identifying spiders at the species level in the genus *Tegenaria* is difficult without using invasive methods (Bolzern, Burckhardt & Hänggi, 2013). *Chilostoma planospira* is a snail that feeds on vegetal organic matter and even lichens (Albano et al., 2014; Baur et al., 2007), while the genus *Limax* contains large slugs (*Limax* were only identified to genus) that feed on residual organic matter (Horsák, Zelený & Hájek, 2014). The frog *Rana italica* and the toad *Bufo bufo* frequently exploit caves but mostly use these habitats as shelter (Bonini, Razzetti & Barbieri, 1999; Lunghi, Manenti & Ficetola, 2014).

### Statistical analyses

The detection of species is imperfect, and detection probability <1 can bias the results of standard regression analyses (MacKenzie et al., 2006). Approaches have been developed to take this into account in regression models (MacKenzie et al., 2006), but they generally require multiple surveys being performed within periods without changes due to birth, death, and movement of individuals to and from the population (closed populations; MacKenzie et al., 2006). Considering the time interval between most of surveys, the assumption of closed populations was likely not met (MacKenzie et al., 2006), making the application of these methods difficult. We therefore used two complementary approaches to control for the potential effects of imperfect detection. First, caves were surveyed using a standardized study design to limit variation in detection rate among sectors, and we dedicated the same sampling effort to all sectors during all seasons. Under standardized monitoring efforts, analyses taking and not taking into account imperfect detection generally produce consistent results (Banks-Leite et al., 2014). We thus analyzed data with generalized linear mixed models (GLMMs). This approach allows for heterogeneity of species distribution among caves and sectors, even though it does not integrate imperfect detection. Subsequently, we repeated analyses using approaches allowing to integrate imperfect detection, to assess the robustness of GLMMs analyses.

### Analysis with mixed models

We identified the relationship between species and cave features using GLMMs assuming binomial error. The presence of each species was considered as a dependent variable while cave features (microclimatic and morphological) were considered as independent variables. As each cave was visited 12 times, cave and sector identity were used as random factors, while month of survey was included as a categorical variable. To test if species tend to frequent different microclimatic zones during a given time, we also considered the interaction between month (sampling period) and microclimatic features as independent variables. All possible combinations of independent variables were built and ranked using the Akaike’s Information Criterion corrected for small sample size (AICc) (Fang, 2011). For each species, we first built GLMMs using all possible combinations of independent variables and the model with the lowest AICc values was considered the best AICc model. Complex models showing AICc values greater than simpler, nested models were not considered in the set of candidate models (Richards, Whittingham & Stephens, 2011). Variables were transformed using logarithms or square root to better fit the normal distribution when necessary. Likelihood ratio tests were used to assess the significance in terms of the best AICc models.

### Analyses taking into account imperfect detection

Species tend to move across sectors during different periods of the year because of the change of microhabitats (see results). However, analyses integrating detection probability generally assume that multiple surveys are performed during short periods in which occupancy is constant (MacKenzie et al., 2006). We therefore used occupancy modelling to calculate the detection probability for each species, on the basis of pairs of surveys that were performed within 14 days, as these periods may better ensure meeting the assumption of closed populations (MacKenzie et al., 2006). To calculate detection probability of species we used 35 pairs of surveys, performed in all caves and across seasons within an interval which did not exceed 14 days. Subsequently, given that we generally performed only one survey per month, we analyzed data using standard general linear models, while weighing absences on the basis of the detection probability of species (Gómez-Rodríguez et al., 2012). Such an approach allows us to integrate imperfect detection even when only one survey per period is performed (Gómez-Rodríguez et al., 2012).

Integrating random factors into this analysis is not possible, thus, cave identity was included as a fixed factor. For each species, we built GLMs with all the possible combinations of independent variables, and ranked them following AICc. We tested the significance of variables included in the best-AICc model using a likelihood ratio test (Bolker et al., 2008).

### Species richness

To analyze the effect of seasonality on species richness, we used GLMMs with Poisson error. In addition to the eight focal taxa, we also integrated data on the distribution of two species that were frequently observed in the caves, but were not considered in single-species analyses: the salamander *Hydromantes italicus* and the spider *Amaurobius ferox*. The salamander *Hydromantes italicus* was the focus of a previously published study performed in the same area (Lunghi, Manenti & Ficetola, 2015) and represents one of the top predators of the studied taxa. The spider *Amaurobius ferox* was recorded during surveys, the number of detections (mean: 3.67 detections per month) was too small for building robust distribution models. Species richness calculated for each sector represented the dependent variable, while microclimatic features (temperature, humidity and light) and their relative interaction with the sampling period (month) were the independent variables. The individual caves and their sectors were used as random factors. All statistical analyses were performed with program R using packages lme4, MuMIn, MASS and unmarked (Bartoń, 2016; Bates et al., 2015; Fiske & Chandler, 2011; R Core Team, 2016; Venables & Ripley, 2002).

## Results

A total of 1,918 observations of the eight focal taxa were recorded among the 16 caves (and 125 cave sectors) during the 12-month study. The most frequent species was *Metellina merianae*, followed by *Tegenaria* sp*.*, and *Meta menardi*. Less frequent species were *Dolichopoda laetitiae*, *Chilostoma planospira* and *Limax* sp. The other two species *Rana italica* and *Bufo bufo* were less common. Observations of taxa are summarized in Table S2. Per-visit detection probability of species ranged between 0.44 and 0.73 (Table S2). Correlation between microclimatic variables was weak (for all comparisons, |*r*| < 0.11), indicating that collinearity is not a problem for our models.

### (a) Species distribution

**Figure 1:**
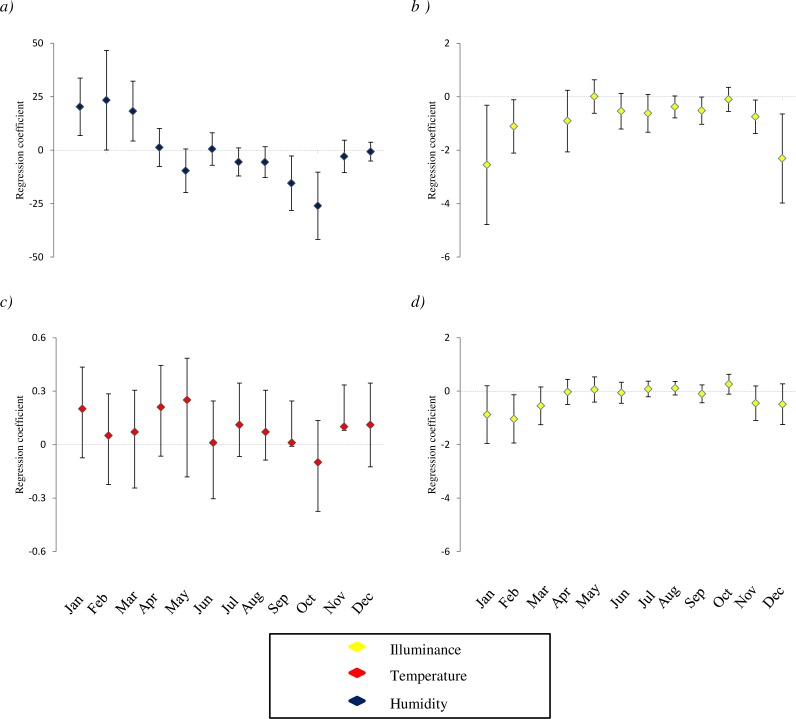
Variation of relationship between the presence of species and microclimatic features. The study species are: (A) *D. laetitiae*, (B and C): *M. menardi*; (D): Tegenaria. For each microclimatic feature (illuminance, temperature and humidity) we show the coefficients of regression models analyzing the different months separately. Positive values indicate that in a given month the species is positively associated with the variable, and so on. Analyses are limited to species by variable combinations for which a significant interaction between month and the variable is included in both GLMMs and GLM best AICc model (see Table 1). Missing values correspond to months in which the models showed convergence issues, due to the limited sample size; error bars are two standard errors.

The distribution of species varied among cave sectors over time. For all taxa, we detected significant relationships between occurrences, survey period, and the environmental features recorded (Table 1), but these relationships were highly variable amongst species. For all taxa, results of both mixed models and GLMs considering imperfect detection were generally consistent, although some relationships were detected by just one of these analyses. The occurrence of the cricket *D. laetitiae* was negatively related to sector height and light, and positively related to wall heterogeneity and temperature. The best AICc models also included the interactions between month and humidity (Table 2A; Table S3A). From winter to early spring this cricket occupied sectors characterized by high humidity, while in late summer and autumn it was associated with less humid sectors (Fig. 1A). Furthermore, the best mixed model also included the interaction between month and light (Table 2A), while the best model considering imperfect detection also included a positive relationship with width of sector and the interaction between month and temperature (Table S3A).

**Table 1:** The best five models based on AICc relating the presence of each taxon. Presence of species (A–H) were considered as dependent variables. Independent variables are: wall heterogeneity (Het), humidity (Humid), Month of survey, minimum illuminance (Lux), temperature (Temp), Height and Width of sectors. Interaction (×) between Month (M) and microclimatic features were considered as further independent variables. The symbol X indicates the presence of variables into models.

Independent variables included into the model										*df*	AICc	Δ-AICc	Weight
**Het**	**Humid**	**Month**	**Height**	**Width**	**Lux**	**Temp**	**Hum**× **M**	**Lux**× **M**	**Temp**× **M**				
**(A) * Dolichopoda laetitiae***													
**X**	**X**	**X**	**X**		**X**	**X**	**X**	**X**		**41**	**889**	**0**	**0.628**
X	X	X	X	X	X	X	X	X		42	890.5	1.5	0.297
X	X	X	X		X	X	X	X	X	52	895.2	6.20	0.028
X	X	X	X	X	X	X	X	X	X	53	896.4	7.43	0.015
X		X	X		X	X			X	29	898.3	9.45	0.006
**(B) *Meta menardi***													
	**X**	**X**	**X**	**X**	**X**	**X**		**X**	**X**	**41**	**1224.1**	**0**	**0.344**
X	X	X	X	X	X	X		X	X	42	1226.1	2.03	0.125
	X	X	X		X	X		X	X	40	1226.5	2.41	0.103
X	X	X			X	X		X	X	40	1227.4	3.33	0.065
		X	X	X	X	X		X	X	40	1227.5	3.43	0.062
**(C) *Metellina merianae***													
		**X**	**X**		**X**	**X**		**X**		**28**	**1424.7**	**0**	**0.174**
X		X			X	X		X		28	1425.2	0.55	0.133
		X		X	X	X		X		28	1425.6	0.88	0.112
	X	X	X		X	X		X		29	1426.4	1.72	0.074
		X	X	X	X	X		X		29	1426.7	2.04	0.063
**(D) *Tegenaria sp.***													
		**X**		**X**	**X**	**X**		**X**		**28**	**1256.1**	**0**	**0.107**
				X	X	X				6	1256.8	0.69	0.076
	X	X		X	X	X		X		29	1257	0.91	0.068
		X	X		X	X		X		28	1257.1	1.01	0.065
X		X			X	X		X		28	1257.5	1.35	0.055
**(E) *Chilostoma planospira***													
		**X**	**X**	**X**	**X**					**17**	**868.3**	**0**	**0.072**
		X	X	X	X	X				18	868.9	0.61	0.053
		X	X	X	X			X		28	869	0.78	0.049
X		X	X		X					17	869.2	0.96	0.045
			X	X	X					7	869.4	1.16	0.040
**(F) *Limax sp.***													
**X**		**X**		**X**						**16**	**703**	**0**	**0.061**
X		X								15	703.1	0.06	0.059
		X	X							15	703.1	0.07	0.059
		X		X						15	703.6	0.63	0.045
		X								14	703.7	0.65	0.044
**(G) *Rana italica***													
**X**		**X**	**X**	**X**	**X**			**X**		**29**	**526.4**	**0**	**0.244**
X		X	X	X	X					18	526.7	0.35	0.205
X		X	X	X	X	X				19	527.7	1.29	0.128
X		X	X	X	X	X		X		30	528.1	1.73	0.103
X	X	X	X	X	X			X		30	528.4	2.08	0.086
**(H) *Bufo bufo***													
**X**			**X**			**X**				**6**	**200.3**	**0**	**0.109**
			X			X				5	200.4	0.08	0.105
X			X		X	X				7	200.5	0.17	0.1
			X	X		X				6	201.1	0.79	0.073
			X		X	X				6	201.4	1.06	0.064

Within the arachnofauna, occurrence of *M. menardi* was positively related with sector width, and negatively with height and light. Interactions between month and light and between month and temperature were also included in the best AICc model (Table 2B; Table S3B). From late summer to winter this spider occupied sectors with warmer temperatures, while sectors with low light were preferred during the whole year (Figs. 1B and 1C). Furthermore, the best mixed model also included a positive relationship with sector humidity (Table 2B). The frequency of *M. menardi* was higher in autumn. The occurrence of *M. merianae* was positively related with sector temperature; the highest frequencies of the species were detected between spring and autumn (Table 2C; Table S3C). The best mixed model also suggested a negative relationship with sector height and light, and detected an interaction between month and light (Table 2C), while the best model considering imperfect detection also included a negative relationship with humidity (Table S3C). The occurrence of *Tegenaria* was negatively related to light. Furthermore, there was a significant interaction between month and light (Table 2D; Table S3D), as the relationships between these spiders and low light was particularly evident in winter (Fig. 1D), a period in which the species was particularly frequent in caves. Furthermore, the best mixed model also included a positive relationship with sector temperature and a negative one with width (Table 2D), while the best GLM also included a negative relationship with sector humidity and height, and the interaction between humidity and month (Table S3D).

**Table 2:** Parameters related to the presence of species. For each species are shown significance of variables included in the relative best AICc model. First eight species (A–H) are the species included in this study, while data for the last species (*Hydromantes italicus*) are taken from another study performed in the same area (Lunghi, Manenti & Ficetola, 2015). Shaded variables are those included in the best model of the same species identified by GLM (only for species studied in this work).

Factor	*B*	${\chi }_{1}^{2}$	*P*
**(A) *D. laetitiae***			
Month		99.91	**<0.001**
Heterogeneity	0.40	13.25	**<0.001**
Height	−0.42	14.68	**<0.001**
Humidity	7.79	0.01	0.919
Lux	−25.13	24.68	**<0.001**
Temperature	0.56	35.07	**<0.001**
Hum × Month		40.26	**<0.001**
Lux × Month		40.49	**<0.001**
**(B) *M. menardi***			
Month		65.36	**<0.001**
Width	0.13	4.52	**0.033**
Height	−0.18	6.29	**0.012**
Humidity	3.71	5.55	**0.018**
Lux	−2.3	31.42	**<0.001**
Temperature	−0.07	0.93	0.334
Tem × Month		33.72	**<0.001**
Lux × Month		34.89	**<0.001**
**(C) *M. merianae***			
Month		19.23	0.057
Height	−0.03	10.79	**0.001**
Lux	−3.14	226.73	**<0.001**
Temperature	0.13	14.57	**<0.001**
Lux × Month		33.72	**<0.001**
**(D) *Tegenaria sp.***			
Month		15.17	0.175
Width	−0.1	17.64	**<0.001**
Lux	−1.17	28.62	**<0.001**
Temperature	0.11	7.44	**0.006**
Lux × Month		30.59	**0.001**
**(E) *C. planospira***			
Month		62.69	**<0.001**
Height	0.11	5.45	**0.019**
Width	−0.13	6.36	**0.012**
Lux	−0.27	6.06	**0.014**
**(F) *Limax sp.***			
Month		91.78	**<0.001**
Heterogeneity	0.15	2.67	0.102
Width	−0.12	2.11	0.147
**(G) *R. italica***			
Month		33.96	**<0.001**
Heterogeneity	−4.62	17.73	**<0.001**
Width	0.34	7.34	**0.007**
Height	0.28	12.90	**<0.001**
Lux	−27.5	11.06	**<0.001**
Lux × Month		23.09	**0.017**
**(H) *B. bufo***			
Heterogeneity	−0.53	2.09	0.148
Height	0.48	15.64	**<0.001**
Temperature	−0.19	5.04	**0.025**
**(I) *H. italicus***			
Month		140.2	**<0.001**
Humidity	−2.65	4.3	**0.039**
Lux	−20.79	7.6	**0.006**
Temperature	0.25	1.4	0.238
Hum × Month		30.6	**0.001**
Temp × Month		31.2	**0.001**

The interaction between month and microclimatic features was never included in the best-AICc model of gastropods, while the month of survey was always included (Tables 2E and 2F). The occurrence of *C. planospira* was positively related with sector height (Table 2E; Table S3E) and occurrences were higher during spring and summer. The best mixed models also included a negative relationship with sector width and light (Table 2E). For *Limax* slugs we did not detect any relationship with the examined cave features, however, its occurrence was higher during summer and early autumn (Table 2F; Table S3F).

The occurrence of *Rana italica* was negatively related to light and wall heterogeneity, and positively related to sector width and height (Table 2G ; Table S3G). The best mixed model also included the interaction between month and light (Table 2G), while the best model considering imperfect detection included a negative relationship with sector humidity (Table S3G). Finally, the occurrence of *B. bufo* was positively related to sector height (Table 2H; Table S3H); the best mixed model included also a negative relationship with temperature (Table 2H).

In this study, we did not include cave depth as an additional variable in models assessing species distribution, because distance from the entrance was strongly related to multiple features of caves, such as light and humidity, and including strongly correlated variables into regression models can bias the regression outcomes (Dormann et al., 2013), making model selection particularly hard (Cade, 2015). For instance, the negative correlation between sector and incident light was very strong (*r* =  − 0.94). However, distance from cave entrance often affects the distribution of non-trogblobitic species (Lavoie, Helf & Poulson, 2007), thus we repeated analyses adding distance from the entrance to the best AICc-models. Adding distance reduced the AICc values of the best models of all species except *Bufo bufo*, and the relationship between depth and species occurrence was negative for most of taxa (Table S5). This suggests that the majority of study taxa are actually found nearby the cave entrance. Nevertheless, adding cave depth to our models did not modify the coefficients of relationships between species and microhabitat features (Table S5), therefore our conclusions are not affected by the decision to not include depth in regression models.

###  (b) Species richness

**Figure 2:**
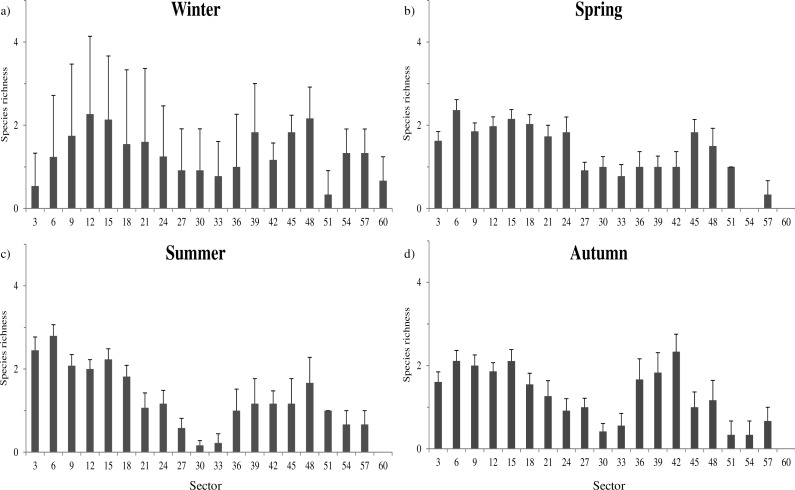
Seasonal variation of species richness. Average species richness estimated for each sector during the whole year 2013. Winter: January–March; Spring: April–June; Summer: July–September; Autumn: October–December. Error bars represent standard errors.

Species richness was mainly related to microclimatic features (Table S4). The richest communities were positively related to sector humidity (${\chi }_{1}^{2}=9.7$, *P* = 0.002) and temperature (${\chi }_{1}^{2}=98.3$, *P* < 0.001), while negatively related with light (${\chi }_{1}^{2}=62.8$, *P* < 0.001). These relationships determined a shift of the sectors with highest richness through the year. During winter, species richness was more evenly distributed among all cave sectors (Fig. 2A), while in spring and summer the highest richness tended to be more concentrated toward the cave entrance (Figs. 2B–2C).

## Discussion

### Species distributions

All the species tend to occupy areas in which their physiological requirements are satisfied (Kearney & Porter, 2009), thus they are associated with areas where the environmental conditions (e.g., microclimate) are suitable. Survey month was a major factor determining the presence of species in caves, as it was present in nearly all best models (Table 2 and Table S3). This fact underlines the high seasonality of species distributions, even for populations exploiting subterranean habitats. In the extreme case of *Limax* slugs, month was the only variable included in the best AICc model (Table 2F). These slugs did not show any significant relationship with the considered microhabitat features, but the occurrence of these gastropods was strongly affected by period of the year. For nearly all the species, the highest occurrence was detected during spring and summer months, periods in which ectothermal animals are generally more active, and food availability is high (Bale & Hayward, 2010).

Physical and morphological cave features (e.g., width, height and wall heterogeneity of inner environment) were very important, especially for invertebrate predators. *Meta menardi* showed an association with sectors characterized by a low cave roof. This predator hunts through both active search and sit-and-wait strategies, so constructing its web in sectors where the ceiling is not far from the ground might increase rates of prey capture in webs. However, cave morphology did not only influence predators. Non-predator species could also be influenced by sector structure, such as *Dolichopoda laetitiae* and *Chilostoma planospira*. The cricket *D. laetitiae* was associated with sectors characterized by high irregularity, and many clefts which may have given the opportunity to better avoid predators. *Chilostoma planospira* was related to sectors in which food availability might be higher, as this species frequented areas characterized by high passage which in turn have more wall surface, as this feature is positively related with the abundance of lichens (Manenti, 2014).

The occurrence of seven out of eight study taxa was significantly influenced by the month of survey; the only exception was *Bufo bufo*. This widespread toad has a wide physiological tolerance, thus, the limited microclimatic excursion of cave environments may have a limited effect on the presence of this species. Furthermore, in three studied taxa we detected clear interactions between month and microclimatic features (Table 2, Fig. 1). These interactions may occur for several reasons. In some cases the species may select the same microhabitat across the seasons and mitigate within the caves, selecting areas with suitable microclimate over the year. For instance, a previous study on the same group of caves showed that cave salamanders are consistently associated with sectors having a temperature of 10−15 °C (Lunghi, Manenti & Ficetola, 2015); in summer these conditions represent the coolest sector, while in winter these are the conditions of warmest sectors. As a consequence, the relationship between salamander occurrence and temperature is positive during winter and negative during summer (Lunghi, Manenti & Ficetola, 2015). However, the significant interaction between environmental variables and month suggests taxa were associated with different microhabitats during different periods. Species were associated with the darkest or most humid sectors in winter, while in summer they were found in superficial sectors with more light and less humidity (Fig. 1). Warmer sectors were more often inhabited during winter (Fig. 1). For most of the species, activity peaks are reached in summer, when individuals are generally closer to the surface. Conversely, during winter periods, species become less active and tend to occupy deeper sectors of caves, where prolonged suitable conditions may let them reduce costs linked to acclimatization (Angilletta Jr, Niewiarowski & Navas, 2002) (Fig. 1).

Our study shows that at least for the populations considered, cave exploitation is not random and occurs during the whole year. Moreover, at least for the relatively superficial cave sectors, the distribution of species is strongly related to the different cave microclimatic features, and to their seasonal variation during the year. Seasonal effects are extremely important, and are a major driver of species distribution (Sheldon & Tewksbury, 2014). In caves, the seasonality occurring in shallow sectors is generally higher than in the deepest parts, where temperature shows very limited variation (Ravbar & Kosutnik, 2014). Nevertheless, even at the cave entrance microclimatic conditions are significantly more stable than in outdoor environments (Ray, Beever & Rodhouse, 2016).

### Species richness

During the year, the highest species richness was found in sectors with specific microclimatic conditions (i.e., high humidity, warm temperature and low light). These conditions are usually realized in sectors that are not far from the entrance, but are deep enough to prevent excessive light exposure (Culver & White, 2005). However, the cave area in which such a combination is realized is not static, thus its location is not fixed but changes depending on surface environmental influence. For example, the stronger the external influence, the deeper the suitable area. In such zones, individuals are protected from extreme external thermic fluctuations but, when external condition are suitable, they are able to reach external foraging sites (Fang, 2011). Distance from the entrance is certainly important for the distribution of most of study species (Table S5), but it is strongly related to the microclimatic features, which actually explain most of variation in species distribution (Table S5). Therefore, we did not include distance as predictor in our models, given that focusing on microclimatic features allows to better characterize the most likely determinant of species

The distribution of sectors with the highest species richness seems to be bimodal, with peaks of richness at about 15 and 48 m from the entrance (Fig. 2).The farther peak of richness might be due to the presence of cryptic access to the main cave environment. In cave studies the main entrances (where humans can get in) are generally considered to be the sole accesses to inner environments. However, even if that is true for humans, small animals can use small fractures and passages to enter cave environments. In fact, in the two longest examined caves, respectively at 48 and 57 m of depth, the presence of interstitial openings likely permits species to enter and exit from cave environments without passing through the main entrance.

### Study limitations

In our study we standardized surveys in order to minimize the impact of variation of detection probability on our results (Banks-Leite et al., 2014). Nevertheless the difficulty of applying occupancy modeling to our data can complicate data interpretation. Indeed, in the results it is not easy to distinguish between true variation in occupancy and variation in detection probability.

To confirm the appropriateness of results, we repeated analyses by integrating the detection probability of each species. The outcome confirmed that mixed model were generally robust, as 70% of relationships detected by mixed models were confirmed by analyses considering imperfect detection. When sites are surveyed only once per period, weighted GLMs can allow us to take into account imperfect detection (Gómez-Rodríguez et al., 2012). However, this approach also has some limitations. Given that standard implementations do not allow including random factors, it hampers our ability to fully consider the effects of spatial heterogeneity, and the potential impacts of cave and sector identity on species distribution. Under these situations, a comparison between the two methods can be the best approach to identify the most consistent relationships, thus obtaining more robust results.

## Conclusion

Cave communities are often considered to be less affected by seasonality than communities from the surface. The species surveyed in our study belong to two different types of nonstrictly cave dwelling organisms: usually epigean species normally considered to exploit caves during certain part of the year or during part of the day, and species resident close to cave entrances (e.g., *Meta* and *Metellina* spiders). Our study also shows that the limited seasonal variation occurring from shallow subterranean habitats to first part of the deep underground environment is strong enough to modify the distributions of nonstrictly cave dwelling species. Indeed, cave environments are not simple refuge for these taxa, but they actually are habitats with dynamic features. This stresses the complexity of interactions between outdoor and subterranean environments. As a consequence, studies on the ecology of cave environments should take into account the dynamism of nonstrictly cave-dwelling species as an active seasonal feature. Future studies are needed to understand the spatial extent of such seasonal influences.

##  Supplemental Information

10.7717/peerj.3169/supp-1 Table S1 Raw data Precise coordinates of caves will be given only under motivated request, as these environments host high density of strictly protected species.

10.7717/peerj.3169/supp-2 Table S2 Detection probability of studied species and their frequencies in cave

10.7717/peerj.3169/supp-3 Table S3 Parameters related to the presence of species (analysis with GLMs taking into account imperfect detection) For each species are shown significance of variables included in the relative best AICc model. Shaded variables are those included in the best model of the same species identified by GLMMs.

10.7717/peerj.3169/supp-4 Table S4 Best 3 AICc models describing community richness The dependent variable is species richness calculated for each sector; the candidate independent variables include microclimatic features (temperature, humidity and illuminance) and their relative interaction with sampling period (month). When a continuous variable is included in the model we showed the regression coefficient, while the symbol + indicate the presence of categorical variable. Cave and sector identity were included as random factors in all the models.

10.7717/peerj.3169/supp-5 Table S5 Generalized linear models (GLMM) also including depth of the sector as a further independent variable Models were built by adding sector depth to the best AICc models in Table 2. We only show the model including depth having AICc values lower than the model not including depth (Table 2).

## Additional Information and Declarations

### Data Availability

The following information was supplied regarding data availability: The raw data has been supplied as a Supplementary File.
