# Supplementary material for: Cave features, seasonality and subterranean distribution of non-obligate cave dwellers
Source: PeerJ. 2017 May 10;5:e3169. doi: 10.7717/peerj.3169 (PMC5428323; doi:10.7717/peerj.3169)
Supplement: Table S2 [file peerj-05-3169-s002.doc]

**Supplementary material**

Table S2: Detection probability of studied species and their frequencies in cave. Detection probability represent the probability to detect at least one individual of the species during one survey; sectors are sections of three meters along the development of cave environments.

| **Species** | **Detection probability** | **Occupied sectors** |
| --- | --- | --- |
| *Meta menardi* | 0.731 | 26.4% |
| *Metellina merianae* | 0.462 | 28.1% |
| *Tegenaria sp.* | 0.586 | 27.3% |
| *Dolichopoda laetitiae* | 0.647 | 19.1% |
| *Chilostoma planospira* | 0.459 | 12.3% |
| *Limax sp.* | 0.516 | 9.3% |
| *Bufo bufo* | 0.444 | 1.9% |
| *Rana italica* | 0.533 | 6.1% |
