# Supplementary material for: Cave features, seasonality and subterranean distribution of non-obligate cave dwellers
Source: PeerJ. 2017 May 10;5:e3169. doi: 10.7717/peerj.3169 (PMC5428323; doi:10.7717/peerj.3169)
Supplement: Table S3 — For each species are shown significance of variables included in the relative best AICc model. Shaded variables are those included in the best model of the same species identified by GLMMs. [file peerj-05-3169-s003.docx]

**Supplementary material**

Table S3: Parameters related to the presence of species (analysis with GLMs taking into account imperfect detection). For each species are shown significance of variables included in the relative best AICc model. Shaded variables are those included in the best model of the same species identified by GLMMs.

| Factor | *B* | χ^2^_1_ | *P* | Factor | *B* | χ^2^_1_ | *P* |
| --- | --- | --- | --- | --- | --- | --- | --- |
| a) *D. laetitiae* |  |  |  | e) *C. planospira* |  |  |  |
|  |  |  |  |  |  |  |  |
| Month |  | 73.79 | **< 0.001** | Month |  | 15.29 | 0.169 |
| Cave |  | 259.29 | **< 0.001** | Cave |  | 194.84 | **< 0.001** |
| Heterogeneity | 0.47 | 12 | **< 0.001** | Heterogeneity | -0.14 | 3.58 | 0.058 |
| Width | 0.28 | 7.95 | **0.005** | Height | 0.12 | 5.36 | **0.02** |
| Height | -0.81 | 21.41 | **< 0.001** | Temperature | 0.09 | 2.73 | 0.098 |
| Humidity | 6.64 | 2.44 | 0.118 |  |  |  |  |
| Lux | -1.26 | 43.99 | **< 0.001** | f) *Limax sp.* |  |  |  |
| Temperature | 0.26 | 20.65 | **< 0.001** |  |  |  |  |
| Temp × Month |  | 43.21 | **< 0.001** | Month |  | 42.39 | **< 0.001** |
| Hum × Month |  | 22.26 | **0.022** | Cave |  | 76.74 | **< 0.001** |
|  |  |  |  |  |  |  |  |
| b) *M. menardi* |  |  |  | g) *R. italica* |  |  |  |
|  |  |  |  |  |  |  |  |
| Month |  | 48.06 | **< 0.001** | Month |  | 26.95 | **0.005** |
| Cave |  | 309.49 | **< 0.001** | Cave |  | 147.15 | **< 0.001** |
| Width | 0.18 | 7.44 | **0.006** | Heterogeneity | -4.10 | 7.70 | **0.005** |
| Height | -0.21 | 6.95 | **0.008** | Width | 0.60 | 23.02 | **< 0.001** |
| Lux | -2.28 | 42.92 | **< 0.001** | Height | 0.29 | 13.22 | **< 0.001** |
| Temperature | -0.14 | 0.61 | 0.433 | Humidity | -5.48 | 7.44 | **0.006** |
| Temp × Month |  | 43.13 | **< 0.001** | Lux | -0.54 | 10.32 | **0.001** |
| Lux × Month |  | 33.56 | **< 0.001** |  |  |  |  |
|  |  |  |  | h) *B. bufo* |  |  |  |
| c) *M. merianae* |  |  |  |  |  |  |  |
|  |  |  |  | Month |  | 11.64 | 0.391 |
| Month |  | 15.21 | 0.173 | Cave |  | 83.151 | **< 0.001** |
| Cave |  | 147.08 | **< 0.001** | Height | 0.14 | 6.35 | **0.012** |
| Humidity | -1.71 | 3.05 | 0.081 |  |  |  |  |
| Temperature | 0.16 | 20.43 | **< 0.001** |  |  |  |  |
|  |  |  |  |  |  |  |  |
| d) *Tegenaria sp.* |  |  |  |  |  |  |  |
|  |  |  |  |  |  |  |  |
| Month |  | 21.50 | **0.028** |  |  |  |  |
| Cave |  | 247.78 | < 0.001 |  |  |  |  |
| Height | -0.12 | 4.37 | **0.036** |  |  |  |  |
| Humidity | -11.70 | 37.74 | **< 0.001** |  |  |  |  |
| Lux | -1.57 | 23.33 | **< 0.001** |  |  |  |  |
| Hum × Month |  | 33.32 | **< 0.001** |  |  |  |  |
| Lux × Month |  | 35.35 | **< 0.001** |  |  |  |  |
