# Supplementary material for: Cave features, seasonality and subterranean distribution of non-obligate cave dwellers
Source: PeerJ. 2017 May 10;5:e3169. doi: 10.7717/peerj.3169 (PMC5428323; doi:10.7717/peerj.3169)
Supplement: Table S4 — The dependent variable is species richness calculated for each sector; the candidate independent variables include microclimatic features (temperature, humidity and illuminance) and their relative interaction with sampling period (month). When a continuous variable is included in the model we showed the regression coefficient, while the symbol + indicate the presence of categorical variable. Cave and sector identity were included as random factors in all the models. [file peerj-05-3169-s004.doc]

**Supplementary material**

Table S4: Best 3 AICc models describing species richness. The dependent variable is species richness calculated for each sector; the candidate independent variables include microclimatic features (temperature, humidity and illuminance) and their relative interaction with sampling period (month). Within candidate independent variables, when a continuous predictor is included in the model we showed the regression coefficient, while the symbol + indicate the presence of categorical variable. Cave and sector identity were included as random factors in all the models.

| **Independent variables included into the model** | | | | | | | **df** | **AICc** | **Δ-AICc** | **weight** |
| --- | --- | --- | --- | --- | --- | --- | --- | --- | --- | --- |
| **Humid** | **Month** | **Lux** | **Temp** | **Hum** ˟ **M** | **Lux** ˟ **M** | **Temp** ˟ **M** |  |  |  |  |
| 0.52 |  | -0.12 | 0.04 |  |  |  | 7 | 2190.3 | 0 | 0.951 |
|  |  | -0.13 | 0.04 |  |  |  | 6 | 2196.2 | 5.93 | 0.049 |
| 0.75 |  |  | 0.03 |  |  |  | 6 | 2244.4 | 54.10 | 0 |
