# Supplementary material for: Cave features, seasonality and subterranean distribution of non-obligate cave dwellers
Source: PeerJ. 2017 May 10;5:e3169. doi: 10.7717/peerj.3169 (PMC5428323; doi:10.7717/peerj.3169)
Supplement: Table S5 — Models were built by adding sector depth to the best AICc models in Table 2. We only show the model including depth having AICc values lower than the model not including depth (Table 2). [file peerj-05-3169-s005.docx]

Supplementary material

Table S5: Generalized linear models (GLMM) also including depth of the sector as a further independent variable. Models were built by adding sector depth to the best AICc models in Table 2. We only show the model including depth having AICc values lower than the model not including depth (Table 2)..

| Factor | *B* | χ^2^_1_ | *P* | Factor | *B* | | χ^2^_1_ | | *P* | |
| --- | --- | --- | --- | --- | --- | --- | --- | --- | --- | --- |
| a) *D. laetitiae* |  |  |  | e) *C. planospira* |  | |  | |  | |
|  |  |  |  |  |  | |  | |  | |
| Depth | -0.1 | 41.12 |  | Depth | -0.12 | | 25.22 | | **< 0.001** | |
| Month |  | 102.27 | **< 0.001** | Month |  | | 64.26 | | **< 0.001** | |
| Heterogeneity | 0.33 | 9.80 | **0.002** | Height | 0.14 | | 7.30 | | **0.006** | |
| Height | -0.37 | 12.33 | **< 0.001** | Width | -0.17 | | 9.66 | | **0.002** | |
| Humidity | 10.65 | 1.09 | 0.296 | Lux | -0.38 | | 13.21 | | **< 0.001** | |
| Lux | -25.73 | 38.02 | **< 0.001** |  |  | |  | |  | |
| Temperature | 0.57 | 38.09 | **< 0.001** | f) *Limax sp.* |  | |  | |  | |
| Hum × Month |  | 40.5 | **< 0.001** |  |  | |  | |  | |
| Lux × Month |  | 45.97 | **< 0.001** | Depth | -0.03 | | 2.89 | | 0.089 | |
|  |  |  |  | Month |  | | 91.59 | | **< 0.001** | |
| b) *M. menardi* |  |  |  | Heterogeneity | 0.15 | | 2.68 | | 0.102 | |
|  |  |  |  | Width | -0.12 | | 2.16 | | 0.141 | |
| Depth | -0.05 | 22.63 | **< 0.001** |  |  | |  | |  | |
| Month |  | 66.27 | **< 0.001** | g) *R. italica* |  | |  | |  | |
| Width | 0.12 | 3.93 | **0.047** |  |  | |  | |  | |
| Height | -0.18 | 6.08 | **0.014** | Depth |  | | 41.41 | | **< 0.001** | |
| Humidity | 4.64 | 8.63 | **0.003** | Month |  | | 38.82 | | **< 0.001** | |
| Lux | -2.44 | 36.06 | **< 0.001** | Heterogeneity | -3.77 | | 21.39 | | **< 0.001** | |
| Temperature | -0.07 | 1.04 | 0.307 | Width | 0.3 | | 6.71 | | **0.01** | |
| Tem × Month |  | 34.41 | **< 0.001** | Height | 0.32 | | 15.04 | | **< 0.001** | |
| Lux × Month |  | 34.74 | **< 0.001** | Lux | -26.7 | | 25.47 | | **< 0.001** | |
|  |  |  |  | Lux × Month |  | | 25.08 | | **0.009** | |
| c) *M. merianae* |  |  |  |  |  | |  | |  | |
|  |  |  |  |  |  | |  | |  | |
| Depth | -0.08 | 37.03 | **< 0.001** |  |  | |  | |  | |
| Month |  | 18.16 | 0.078 |  |  | |  | |  | |
| Height | -0.04 | 16.75 | **<0.001** |  |  | |  | |  | |
| Lux | -3.36 | 226.73 | **< 0.001** |  |  | |  | |  | |
| Temperature | 0.14 | 14.97 | **< 0.001** |  |  | |  | |  | |
| Lux × Month |  | 35.23 | **< 0.001** |  |  | |  | |  | |
|  |  |  |  |  |  |  |  |  |  |  |
| d) *Tegenaria sp.* |  |  |  |  |  |  |  |  |  |  |
|  |  |  |  |  |  |  |  |  |  |  |
| Depth | -0.13 | 45.39 | **< 0.001** |  |  |  |  |  |  |  |
| Month |  | 15.61 | 0.156 |  |  |  |  |  |  |  |
| Width | -0.11 | 22.32 | **< 0.001** |  |  |  |  |  |  |  |
| Lux | -1.27 | 41.91 | **< 0.001** |  |  |  |  |  |  |  |
| Temperature | 0.12 | 8.70 | **0.003** |  |  |  |  |  |  |  |
| Lux × Month |  | 31.31 | **<0.001** |  |  |  | |  | |  |
